# The degree of equity and coupling coordination of staff in primary medical and health care institutions in China 2013–2019

**DOI:** 10.1186/s12939-021-01572-6

**Published:** 2021-10-30

**Authors:** Weicun Ren, Clifford Silver Tarimo, Lei Sun, Zihan Mu, Qian Ma, Jian Wu, Yudong Miao

**Affiliations:** 1 College of Public Health, Zhengzhou University, 100, Science Avenue, Gaoxin District, Zhengzhou, 450001 Henan China; 2 Department of Health Management, Sanquan College of Xinxiang Medical University, Xinxiang, 453000 Henan China; 3 Dares Salaam Institute of Technology, Department of Science and Laboratory Technology, P.O. Box 2958, Dar es Salaam, Tanzania

**Keywords:** Primary medical and health care institutions, Equity, Coupling coordination degree, Medical staff, GM (1, 1)

## Abstract

**Background:**

Primary medical and health care facilities are the first lines of defense for the health of population. This study aims to evaluate the current state and trend of equity and coupling coordination degree (CCD) of staff in primary medical and health care institutions (SPMHCI) based on the quantity and living standards of citizens in China 2013–2019. The research findings are expected to serve as a guideline for the allocation of SPMHCI.

**Methods:**

The data used in this study including the quantity and living standards of citizens, as well as the number of SPMHCI in 31 provincial administrative regions of China, were obtained from the China Statistical Yearbook and the China Health Statistics Yearbook. The equity and CCD for SPMHCI were analyzed by using the Gini coefficient and the CCD model, and the Grey forecasting model GM (1, 1) (GM) was used to predict the equity and CCD from 2020 to 2022.

**Results:**

Between 2013 and 2019, the number of SPMHCI increased from 3.17 million to 3.50 million, and the population-based Gini coefficient declined from 0.0704 to 0.0513. In urban and rural areas, the Gini coefficients decreased from 0.1185 and 0.0737 to 0.1025 and 0.0611, respectively. The CCD between SPMHCI and citizens’ living standards (CLS) changed from 0.5691, 0.5813, 0.5818 to 0.5650, 0.5634, 0.6088 at national, urban, and rural levels, respectively. The forecasting results of GM revealed that at the national, urban and rural levels from 2020 to 2022, the Gini coefficient would rise at a rate of − 13.53, − 5.77%, and − 6.10%, respectively, while the CCD would grow at a rate of - 0.89, 1.06, and 0.87%, respectively.

**Conclusions:**

In China, the number of SPMHCI has increased significantly, with an equitable allocation based on the population. The interaction between SPMHCI and CLS is sufficient, but the degree of mutual promotion is moderate. The government could optimize SPMHCI and improve the chronic disease management services to improve CLS and to ensure the continued operation of primary medical and health care institutions in urban areas.

**Supplementary Information:**

The online version contains supplementary material available at 10.1186/s12939-021-01572-6.

## Introduction

Primary health care is gaining popularity worldwide since everyone has the right to receive basic health care [1, 2]. Primary medical and health care institutions have gradually evolved into the guardians of people’s health, assuming the functions of first diagnosis, referral, chronic disease management, health education, and post-ill rehabilitation as the hierarchical diagnosis and treatment system has advanced [3, 4]. Human resources for health are a critical component of the development of medical and health care services. As the foundation of the entire medical and health care service system, the development and innovation of primary medical and health care institution determines the quality of primary medical work including diagnosis, treatment, as well as the levels of all basic public health services [5, 6]. In China, the “Healthy China 2030” initiative proposed in 2016 emphasizes that it is necessary to strengthen the grass-roots talent team and increase support for the grass-roots and remote areas [7]. In addition, Guiding Opinions on Further Regulating Community Health Service Management and Improving Service Quality put forward more specific guidelines for the development of health manpower in primary medical and health care institutions [8].

Aging population [9], chronic noncommunicable diseases [10], and total health expenditure [11] are all on the rise, and the role of primary medical and health care institutions is becoming increasingly important. However, the number of primary medical and health care institutions in China in 2019 was 687,731, accounting for 68.26% of all medical institutions. Among them, the number of institutions in urban and rural areas are 35,013 and 652,718, respectively [12]. The staff in primary medical and health care institutions (SPMHCI) is 25.07 per 10,000 citizens [13]. Simultaneously, the SPMHCI continues to face challenges that affect the sustainable development of primary medical and health institutions, including significant losses, unscientific management, irrational organizational structures, and low levels of academic qualifications and professional titles [14]. Therefore, as one of the important factors that affect the management and development of primary medical and health institutions, the equitable and coordinated allocation of human resources deserves special consideration [1].

Studies on the equitable of SPMHCI have provided critical evidence for optimizing resource distribution. Researchers employ a variety of techniques to analyze and assess the equity of staffing in diverse regions and institutions. Efrat S et al. analyzed the role of equity in the allocation of SPMHCI providing preventive services [15]. The study discovered that the distribution of general practitioners in Australia was skewed using the Robin Hood Index [16]. Sun WX discovered that, in terms of Gini coefficient, the primary medical and health institutions in Jilin province in China allocated the health resources more efficiently by population than by geographic area [17]. In addition, Lin CM found that the inequity for the allocation of general practitioners between districts and counties in Beijing had been increasing year by year in terms of maximum/minimum value (multiples), relative difference coefficient, Gini coefficient, and difference index [18].

The coordination between SPMHCI and the health demands of citizens determines the efficiency and effectiveness of their work [19]. Citizens’ health demands are closely related to their standard of living [20]. Miller Fiona A et al. proposed four regulatory coordination models such as health technology assessment organizations and group procurement organizations by using the methods of document review and key informant interviews [21]. Okech M et al. discovered that human resources for health coordination platforms with strong governance structures are sustainable [19], while Wang W examined the interaction between investment in medical staff and urbanization in China from 1995 to 2015 and found that the two systems were in a convergence trend but still show a low coupling strength and a low level of coordination [22]. Zhang C used the entropy weight-set pair analysis model to quantify and evaluate the basic public services and economic development in 16 cities in Anhui Province from 2009 to 2018, revealing a lack of overall coordination between the two [23].

As previously stated, existing research on the equity of SPMHCI is primarily focused on regions classified by administration, economy, and geographic location, with relatively little comparative analysis between urban and rural primary medical and health institutions [3–5]. Additionally, prior research on the relationship between SPMHCI and other facets of society has tended to focus on institutional and economic dimensions, with little attention paid to the level of citizens’ living standards (CLS). We think that it is necessary to adjust and optimize the allocation of SPMHCI in a timely manner in order to promote social equity and meeting the needs of the people. Thus, this study examined the current state, equity, coupling degree and coupling coordination degree (CCD) of SPMHCI in China from 2013 to 2019. The Gini coefficient was used to analyze the equity of SPMHCI based on the population. In addition, the CCD model was used to evaluate the coupling degree and CCD between SPMHCI and CLS at national, urban, and rural levels. Finally, the number, equity, and CCD of SPMHCI in China from 2020 to 2022 were predicted by using the Grey forecasting model GM (1, 1) (GM) in order to further explore the future development of SPMHCI. Findings of the current study are expected to serve as a guide for the government to optimize the allocation of SPMHCI.

## Materials and methods

### Data sources

The data for this study come from 31 provincial-level administrative regions in China, which include 22 provinces, five autonomous regions (Inner Mongolia, Guangxi, Tibet, Ningxia, and Xinjiang), and four municipalities (Beijing, Tianjin, Shanghai, and Chongqing). The China Statistical Yearbook provided information on the quantity of citizens and CLS in each region (2014–2020) [24] while data on the number of SPMHCI was obtained from the China Health Statistical Yearbook (2014–2020) [12, 25–30], and the total sample size is 217. Among them, CLS information includes information on per capita disposable income and per capita consumption expenditure, and SPMHCI refers to the total number of staff in primary medical and health care institutions, including doctors, nurses, management personnel, medical technicians, logistical personnel and others. In this study, primary medical and health care institutions refer to community health service centers and stations in urban areas, as well as township health centers and village clinics in rural areas. The eastern region, central region, and western region of China were defined according to the China Health Statistics Yearbook 2020.

### Data analysis

#### Gini coefficient

The Gini coefficient was proposed by Corrado Gini in 1912, which indicates the income distribution of a country or region and the equity of social wealth distribution [31]. It can also be used to assess the equity of public resource allocation. At the moment, methods for calculating the Gini coefficient are quite developed. Besides the most frequently used method with regional groups based on the Lorentz curve’s interpretation, there are methods using covariance and square difference [32]. Among them, the square difference method is used to analyze data in which the research object cannot be grouped evenly and is less grouped [32]. Given that the data are based on a single province, the population cannot be divided equally into groups, and thus the regional grouping method is not applicable. The covariance method is more accurate for calculations involving a larger number of groups, but China’s provincial administrative regions are relatively few in number. According to the applicability of the methods, this study calculates the Gini coefficient for SPMHCI based on the quantity of citizens using square difference method. In this study, 22 provinces, 5 autonomous regions, and 4 municipalities were ranked based on the number of SPMHCI. The formula for calculating the national and regional Gini coefficients using average difference is:

$$\mathrm{G}=1\hbox{-} \sum \limits_{\mathrm{i}=1}^{\mathrm{n}}{P}_i\left(2{Q}_i-{W}_i\right)$$

$$Q{}_i=\sum \limits_{i=1}^n{W}_i$$

Among them, “n” represents the number of groups; W_i_ represents the ratio of the number of SPMHCI in the i-th group to that of the total in the country; P_i_ denotes the ratio of the quantity of citizens in the i-th group to that of the total population; Q_i_ indicates the cumulative percentage of W_i_ from group 1 to group i; the sum of W_i_ and P_i_ for all groups are both 1 (i = 1, 2, 3, ..., n).

The value of the Gini coefficient is between 0 and 1, and the smaller the value, the higher the equity of SPMHCI [33].

#### Coupling coordination degree model

The role of coupling degree model is to quantify the degree of interaction between systems or various elements within them, indicating whether the movement among elements can achieve benign interactions of two or more parties [34]. Coordination degree aims at quantifying the degree of multi-party cooperation between systems or various elements within the system in a certain development stage. This study analyzed the CCD between SPMHCI and CLS systems to indicate whether the movement trend among two systems can form a law of pulling growth in the same direction during the development process [35]. The formula for calculating the coupling degree between the two systems is as follows:

$${y}_{1i}=\frac{x_{1i}-{x}_{1\min }}{x_{1\max }-{x}_{1\min }},\kern0.5em {y}_{2i}=\frac{x_{2i}-{x}_{2\min }}{x_{2\max }-{x}_{2\min }},\kern0.5em {y}_{3i}=\frac{x_{3\max }-{x}_{3i}}{x_{3\max }-{x}_{3\min }}$$

$${d}_j=1+\frac{\sum \limits_{i=1}^{31}{y}_{ji}\ln {y}_{ji}}{\ln 31},\kern1em {w}_j=\frac{d_j}{\sum \limits_{j=1}^3{d}_j}$$

$${\mathrm{U}}_1=\frac{\sum \limits_{i=1}^{31}{y}_{1i}}{31},{U}_2=\frac{\sum \limits_{i=1}^{31}{y}_{2i}}{31}\ast \frac{w_2}{w_2+{w}_3}+\frac{\sum \limits_{i=1}^{31}{y}_{3i}}{31}\ast \frac{w_3}{w_2+{w}_3}$$

$$\mathrm{C}=\frac{\sqrt{U_1{U}_2}}{\frac{U_1+{U}_2}{2}}$$

Among them, y_1i_, y_2i_, y_3i_, x_1i_, x_2i_, x_3i_, x_1max_, x_2max_, x_3max,_ x_1min_, x_2min_, x_3min_ represent the standardized, original, maximum and minimum values of SPMHCI, per capita disposable income and per capita consumption expenditure, respectively (i = 1, 2, 3, ...,31); d_j_ and w_j_ respectively represent the information utility value and weight of SPMHCI, per capita disposable income and per capita consumption expenditure (j = 1, 2, 3). U_1_ and U_2_ denote the comprehensive evaluation value of SPMHCI and CLS, respectively. C is the coupling degree between the two systems, C∈[0, 1]. The closer the C value is to 1, the more coupling the development of the two systems.

The degree of coupling only reflects the degree of interaction between the systems, not the level of each system [36]. The CCD can not only reflect whether each system has a good level, but also reflect the degree of mutual promotion between systems [35]. To understand the CCD between SPMHCI and CLS, a model is established:

$$\mathrm{D}=\sqrt{\mathrm{C}\times T}$$

$$\mathrm{T}={w}_1{\mathrm{U}}_1+\left({w}_2+{w}_3\right){\mathrm{U}}_2$$

Where “D” denotes the CCD; “C” indicates the coupling degree; “T” denotes the total evaluation value of SPMHCI and CLS.

This study referred to the study conducted by Wang RX [32] and categorized the CCD of SPMHCI and CLS into 6 levels. The CCD value less than 0.5 indicates that SPMHCI and CLS are uncoordinated while the value between 0.5and 0.6, 0.6 and 0.7, 0.7 and 0.8 indicates edge coordination, preliminary coordination and intermediate coordination, respectively. CCD value between 0.8–0.9 indicates good coordination while values between 0.9–1.0 indicates high-quality coordination.

#### Grey forecasting model GM (1, 1)

Grey system theory believes that even an irregular discrete time-space sequence has potential orderliness, which can become a regular sequence that satisfies the grey system theoretical modeling conditions [37]. The use of grey system theory for prediction has the advantages of high accuracy, fewer sample data and simple calculation principle and method. Grey system theory includes four types of models: GM (1, 1), DGM (1, 1), GM (1, N) and Verhulst. The commonly used one is the GM (1, 1) model, which represents a first-order, one-variable prediction model.

Firstly, create an original data column based on the number, equity and CCD data of SPMHCI in China from 2013 to 2019 followed by generating a cumulative sequence based on the original data column. We then tested the quasi-smoothness and quasi-exponential law of the cumulative sequence and finally applied the least square method to solve the model parameters (a, u) based on the cumulative sequence [38]. The final predictive model available is:

$${x}^{(1)}\left(i+1\right)=\left[{x}^{(1)}(1)-\frac{u}{a}\right]{e}^{- au}+\frac{u}{a}$$

Where x^(1)^(i) represents the i-th number in a cumulative sequence (i = 1, 2, 3, ..., 7) and e is the natural index while a, u are parameters.

### Analysis tool

The initial data was collected and processed by using Excel 2019 software. Excel 2019 software was also used to perform Gini coefficient and CCD analysis. GM was performed by using Matlab 2014 software. *P* < 0.05 was considered statistically significant.

## Results

This study analyzed the trend, equity, and CCD for SPMHCI from 2013 to 2019 at the national, urban, and rural levels. The analyzed parameters include the Gini coefficient and CCD. The GM was applied to predict the trend, equity, and CCD from 2020 to 2022.

### The basic situation of SPMHCI in China from 2013 to 2019

In China, the number of SPMHCI increased from 3.17 million in 2013 to 3.50 million in 2019, at an average annual rate of 1.76%. At the end of 2019, the population of China was 1.397 billion. In terms of CLS, average annual growth rates for per capita disposable income and consumption expenditure were 11.31 and 10.51%, respectively. Table 1 and Appendix Table show the detailed information.

**Table 1:** The baseline situation of Chinese citizens and SPMHCI from 2013 to 2019

Index	2013	2014	2015	2016	2017	2018	2019	Average growth rate (%)
**SPMHCI**^**a**^ **(10,000 people)**								
Country	316.72	319.65	320.02	327.86	336.99	341.52	350.09	1.76
Urban	47.61	48.88	50.48	52.20	55.47	58.29	61.03	4.70
Rural	269.11	270.77	269.54	275.66	281.52	283.23	289.06	1.24
**CLS**^**b**^ **in national areas (yuan)**								
Per capita disposable income	18,310.8	20,167.1	21,966.2	23,821.0	25,973.8	28,228.0	30,732.8	11.31
Per capita consumption expenditure	13,220.4	14,491.4	15,712.4	17,110.7	18,322.1	19,853.1	21,558.9	10.51
**CLS in urban areas (yuan)**								
Per capita disposable income	26,955.1	28,843.9	31,194.8	33,616.2	12,363.4	36,396.2	42,358.8	9.52
Per capita consumption expenditure	18,022.6	19,968.1	21,392.4	23,078.9	10,129.8	24,445.0	28,063.4	9.29
**CLS in rural areas (yuan)**								
Per capita disposable income	9429.6	10,488.9	11,421.7	12,363.4	13,432.4	14,617.0	16,020.7	11.65
Per capita consumption expenditure	6625.5	8382.6	9222.6	10,129.8	10,954.5	12,124.3	13,327.7	16.68
**Citizens**^**c**^ **(10,000 people)**								
Country	134,792	135,514	136,249	137,088	137,982	138,834	139,656	0.60
Urban	71,626	73,492	75,385	77,517	79,791	81,972	83,946	2.87
Rural	63,166	62,022	60,864	59,571	58,191	56,862	55,710	−1.97

^a^SPMHCI: Staff in primary medical and health care institutions

^b^CLS: Citizens’ living standards

^c^Citizens: Number of citizens at the end of the year

### The equity for the allocation of SPMHCI

From 2013 to 2019, the population-based Gini coefficient of SPMHCI dropped from 0.0704 to 0.0513, with a rate of change of − 27.13%. In addition, the Gini coefficients of SPMHCI at urban and rural levels were all lower than 0.1207 from 2013 to 2019, and the rate of change was − 13.50% and − 17.10%, respectively. The results also revealed that in China, the allocation of SPMHCI in rural areas was more equitable than that in urban areas. See Table 2.

**Table 2:** Gini Coefficient for allocation of SPMHCI^a^ in China from 2013 to 2019

Variable	2013	2014	2015	2016	2017	2018	2019	Growth rate (%)
National	0.0704	0.0687	0.0646	0.0552	0.0515	0.0487	0.0513	−27.13
Urban	0.1185	0.1207	0.1203	0.1156	0.1102	0.1128	0.1025	−13.50
Rural	0.0737	0.0696	0.0688	0.0615	0.0594	0.0604	0.0611	−17.10

^a^SPMHCI: Staff in primary medical and health care institutions

### The CCD between SPMHCI and CLS

The weighted analysis based on the entropy method found that the change rates of indicator weights of CLS and SPMHCI from 2013 to 2019 were − 19.01 and 17.87%, respectively. Specific to urban and rural areas, the weights of SPMHCI and CLS were 0.3985–0.3055 and 0.6015–0.6945 in urban areas; in rural areas, the weights of SPMHCI and CLS were 0.4014–0.4942 and 0.5058–0.5986, respectively.

Coordination between SPMHCI and CLS in the national, urban, and rural areas were 0.9957–0.9999, 0.9951–0.9999, and 0.9886–0.9999, respectively. The growth rate of coupling degree was negative only in urban areas (− 0.08%). On the other hand, the national level CCD between SPMHCI and CLS from 2013 to 2019 were 0.5642–0.5775, and the maximum CCD in urban and rural areas were 0.5813 and 0.6088, respectively. Only in rural areas did CCD grow at a positive rate (4.64%), which was much higher than the growth rate of CCD at the national and urban areas. For more details, see Table 3.

**Table 3:** The CCD^a^ between SPMHCI^b^ and CLS^c^ in China from 2013 to 2019

Variable	2013	2014	2015	2016	2017	2018	2019	Growth rate (%)
**Weight**								
National								
SPMHCI	0.4846	0.4617	0.4378	0.4277	0.4069	0.3986	0.3925	−19.01
CLS	0.5154	0.5383	0.5622	0.5723	0.5931	0.6014	0.6075	17.87
Urban								
SPMHCI	0.3843	0.3199	0.3174	0.3055	0.3985	0.3235	0.3470	−9.71
CLS	0.6157	0.6801	0.6826	0.6945	0.6015	0.6765	0.6530	6.06
Rural								
SPMHCI	0.4942	0.4577	0.4511	0.4462	0.4565	0.4014	0.4701	−4.88
CLS	0.5058	0.5423	0.5489	0.5538	0.5435	0.5986	0.5299	4.76
**Coupling degree**								
National	0.9957	0.9977	0.9991	0.9999	0.9995	0.9981	0.9977	0.20
Urban	0.9999	0.9963	0.9976	0.9957	0.9951	0.9977	0.9991	−0.08
Rural	0.9886	0.9895	0.9915	0.9932	0.9947	0.9999	0.9971	0.86
**CCD**								
National	0.5691	0.5748	0.5755	0.5775	0.5712	0.5642	0.5650	−0.72
Urban	0.5813	0.5434	0.5455	0.5359	0.5392	0.5443	0.5634	−3.08
Rural	0.5818	0.5953	0.5988	0.6015	0.6000	0.6075	0.6088	4.64

^a^CCD: Coupling coordination degree

^b^SPMHCI: Staff in primary medical and health care institutions

^c^CLS: Citizens’ living standards

### Forecast of the equity and CCD of SPMHCI from 2020 to 2022

**Fig. 1:**
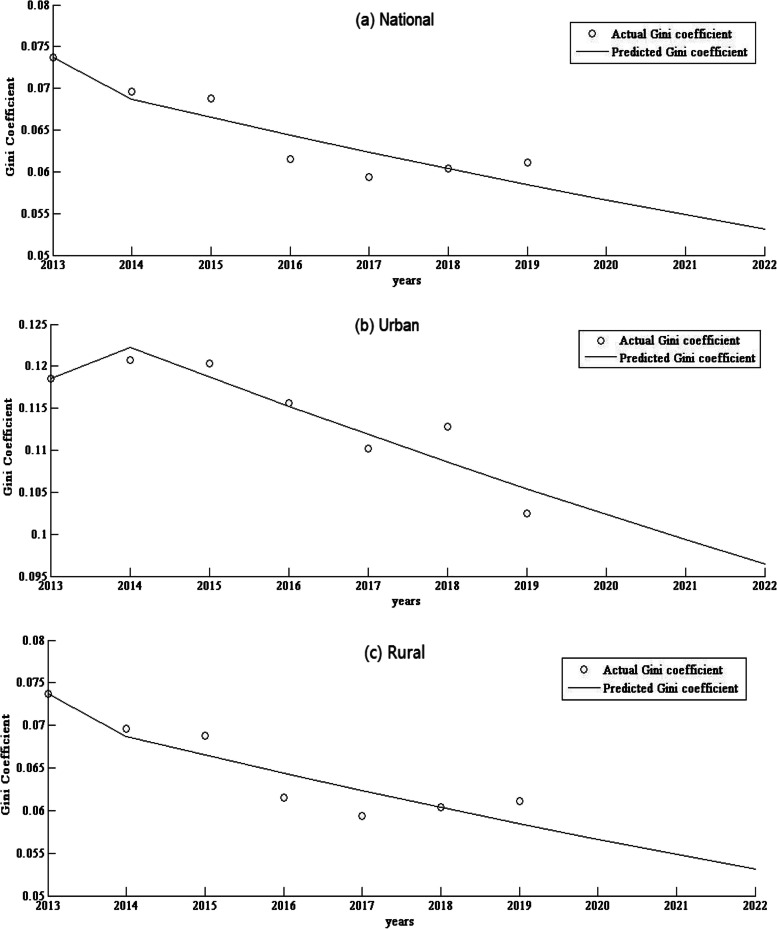
Prediction curve of the Gini coefficient in China’s national, urban and rural areas from 2013 to 2022

**Fig. 2:**
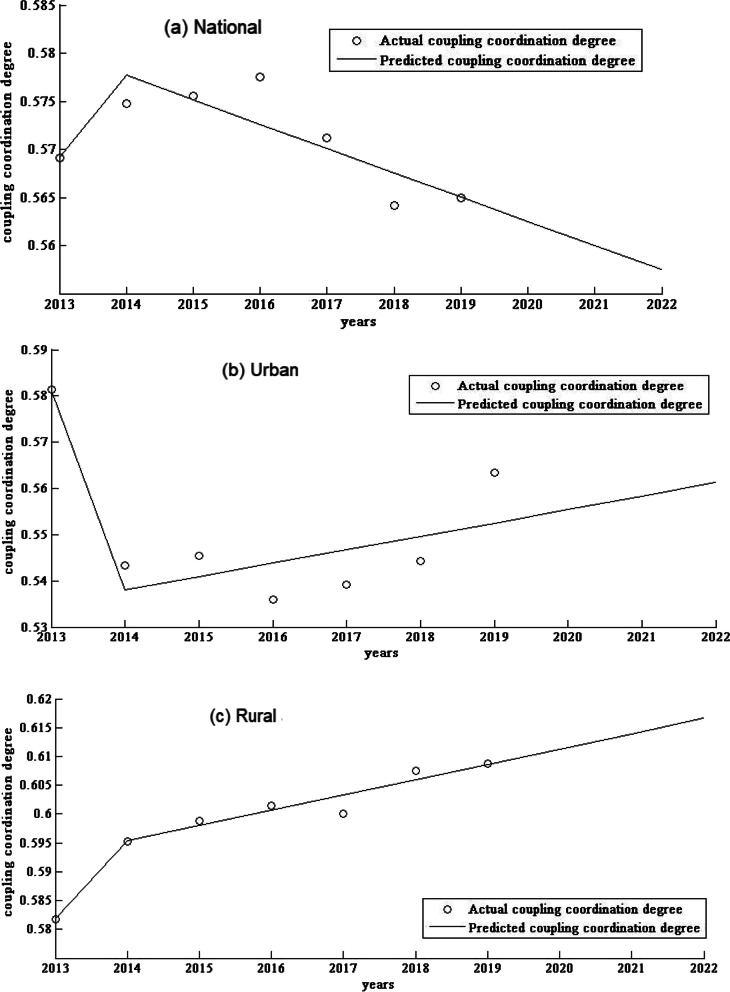
Prediction curve of the coupling coordination degree in China’s national, urban and rural areas from 2013 to 2022

Based on the equity and CCD data of SPMHCI from 2013 to 2019, this study applies GM to forecast of the equity and CCD from 2020 to 2022, and the model fits well with all the variance ratio (VR) less than 0.5. The GM analysis found that population-based Gini coefficients of SPMHCI in 2020, 2021 and 2022 would be 0.0436, 0.0406 and 0.0377, respectively. And the Gini coefficients at urban and rural levels would be 0.1023, 0.0993, 0.0964 and 0.0566, 0.0548, 0.0531, respectively. Between 2020 and 2022, the CCD between SPMHCI and LSR would predict a value of 0.5577–0.5625, with a growth rate of − 0.89%. The growth rates for urban and rural areas are projected to be 1.06 and 0.87%, respectively. See Table 4, Fig. 1 and Fig. 2.

**Table 4:** Prediction of the equity and CCD^a^ of SPMHCI^b^ in China from 2020 to 2022

Variable	2020	2021	2022	Growth rate (%)	Variance ratio
**Gini Coefficient**					
National	0.0436	0.0406	0.0377	−13.53	0.3019
Urban	0.1023	0.0993	0.0964	−5.77	0.3663
Rural	0.0566	0.0548	0.0531	−6.18	0.3973
**CCD**					
National	0.5625	0.5599	0.5575	−0.89	0.5258
Urban	0.5554	0.5584	0.5613	1.06	0.4475
Rural	0.6113	0.6139	0.6166	0.87	0.1737

^a^CCD: Coupling coordination degree

^b^SPMHCI: Staff in primary medical and health care institutions

In addition, we also forecast the number of SPMHCI in China from 2020 to 2022. The predictive analysis results of the GM found that the number of SPMHCI in urban and rural areas in 2020, 2021 and 2022 are projected to be 637,270, 667,420, 698,990 and 2,924,200, 2966,200, 3,008,700, respectively. The rate of change would be 9.69 and 2.89%. More details are displayed in Table 5.

**Table 5:** Prediction of SPMHCI^a^ in China’s urban and rural areas from 2020 to 2022 (people)

Area	2020		2021		2022		Growth rate (%)	
	Urban	Rural	Urban	Rural	Urban	Rural	Urban	Rural
Total	637,270	2,924,200	667,420	2,966,200	698,990	3,008,700	9.69	2.89
Eastern region^b^	359,280	1,012,600	375,620	1,024,200	392,700	1,036,000	9.30	2.31
Middle region^c^	145,300	1,002,300	150,370	1,008,100	155,610	1,013,900	7.10	1.16
Western region^d^	133,000	905,810	142,040	928,230	151,700	951,200	14.06	5.01
Beijing	41,114	4524	43,313	4494	45,629	4464	10.98	−1.33
Tianjin	10,683	13,016	11,304	13,209	11,960	13,405	11.95	2.99
Hebei	20,460	178,990	21,491	180,390	22,573	181,810	10.33	1.58
Shanxi	14,059	80,159	14,489	81,102	14,931	82,056	6.20	2.37
Inner Mongolia	14,798	53,491	15,431	54,710	16,091	55,957	8.74	4.61
Liaoning	20,506	59,297	21,514	59,672	22,571	60,051	10.07	1.27
Jilin	10,047	45,289	10,356	44,656	10,674	44,032	6.24	−2.78
Heilongjiang	14,700	54,370	14,562	54,061	14,426	53,753	−1.86	−1.13
Shanghai	37,331	3577	37,933	3316	38,545	3073	3.25	−14.09
Jiangsu	58,956	18,412	62,418	19,356	66,084	20,349	12.09	10.52
Zhejiang	44,331	88,722	45,430	91,686	46,556	94,749	5.02	6.79
Anhui	21,614	122,260	22,297	123,100	23,002	123,960	6.42	1.39
Fujian	16,025	75,993	17,012	77,181	18,060	78,387	12.70	3.15
Jiangxi	8380	109,470	8325	109,760	8271	110,050	−1.30	0.53
Shandong	44,982	243,290	47,670	238,330	50,519	233,470	12.31	−4.04
Henan	27,717	268,320	29,140	268,060	30,636	267,800	10.53	−0.19
Hubei	26,430	147,390	27,373	148,320	28,350	149,260	7.26	1.27
Hunan	22,771	175,740	24,656	180,200	26,697	184,780	17.24	5.14
Guangdong	61,390	143,650	64,158	146,720	67,051	149,850	9.22	4.32
Guangxi	10,395	115,730	11,253	116,210	12,183	116,700	17.20	0.84
Hainan	3874	19,965	4094	20,610	4327	21,276	11.69	6.57
Chongqing	14,427	62,013	15,398	61,826	16,435	61,639	13.92	−0.60
Sichuan	25,789	212,330	27,115	216,930	28,510	221,620	10.55	4.38
Guizhou	15,788	90,921	18,324	93,337	21,266	95,818	34.70	5.39
Yunnan	11,332	114,490	12,550	123,500	13,898	133,220	22.64	16.36
Tibet	354	19,960	395	20,995	441	22,084	24.58	10.64
Shaanxi	13,028	91,253	13,381	93,136	13,745	95,059	5.50	4.17
Gansu	9973	65,909	10,512	67,176	11,080	68,468	11.10	3.88
Qinghai	3125	16,196	3342	16,561	3575	16,934	14.40	4.56
Ningxia	3624	11,980	4421	12,455	5393	12,949	48.81	8.09
Xinjiang	11,110	53,253	11,562	54,772	12,032	56,333	8.30	5.78

^a^SPMHCI: Staff in primary medical and health care institutions

^b^The eastern region includes Beijing, Tianjin, Hebei, Liaoning, Shanghai, Jiangsu, Zhejiang, Fujian, Shandong, Guangdong, and Hainan

^c^The middle region includes Shanxi, Jilin, Heilongjiang, Anhui, Jiangxi, Henan, Hubei, and Hunan

^d^The western region includes Inner Mongolia, Chongqing, Guangxi, Sichuan, Guizhou, Yunnan, Tibet, Shaanxi, Gansu, Qinghai, Ningxia, and Xinjiang

## Discussion

This study aimed to evaluate the equity and coordination of SPMHCI by analyzing the population-based Gini coefficient and the CCD between CLS in China. The findings show that while the number of SPMHCI has increased significantly in China, it is still insufficient. The population-based Gini coefficient and the coupling degree between CLS were impressive, but the CCD between CLS was somewhat moderate.

Specifically, The Gini coefficient for the allocation of SPMHCI in China dropped from 0.0704 in 2013 to 0.0513 in 2019, which is a more equitable value. China’s SPMHCI allocation plan is mainly based on the standard of health resources per 10,000 population, which is equal in population-based allocation and has shown a more equitable development trend in recent years. However, the Gini coefficient at urban and rural levels from 2013 to 2019 decreased from 0.1185, 0.0737 to 0.1025, 0.0611, respectively. It shows that the equity of SPMHCI at national level is better than those at rural and urban levels. This is consistent with the research results of Kong Y [39] and Liu ZY et al. [40]. The prediction results based on GM are as well consistent with this conclusion.

In comparison to national equity, the poor equity score calculated at the urban and rural levels may be related to the dispersed lifestyles of rural residents in western China, which is why the government has increased the number of SPMHCI to ensure residents have access to basic medical services. At the same time, urban areas pay less attention to SPMHCI than rural areas do, owing to increased allocation of medical resources and availability of choices [41]. However, with the improvement of population aging and the increase of chronic disease patients, the government and heads of urban primary medical and health care institutions should pay greater attention to the allocation of SPMHCI to provide better health guidance, rehabilitation, and nursing services to community citizens.

The results show that the coupling degree between SPMHCI and CLS at the national, urban and rural levels were all greater than 0.98, and the rates of change were 0.20, − 0.08% and 0.86%, respectively. This demonstrates that there is a strong interaction between the two systems. Improvement in CLS can convert more health-care needs into demand, increasing the efficiency, benefit, and effectiveness of SPMHCI’s work. More SPMHCI, on the other hand, will help improve citizens’ disease prevention, health, and labor ability, as well as reduce medical expenses and provide more income, thereby improving CLS. Although the coupling degree is very good, the CCD between SPMHCI and CLS in 2013–2019 was 0.5642–0.5775, indicating that at present, the mutual promotion of SPMHCI and CLS has not been fully exerted.

In addition, the prediction results show that the CCD at the national level is declining but the CCD in urban and rural areas are both on an upward trend. The CCD in rural areas is higher than that in urban, which may be due to the lack of medical resources in rural areas and the greater role of SPMHCI in reducing citizen’s medical expenditure and maintaining their work capacity [21, 42]. The government could fully utilize SPMHCI’s role as gatekeeper in the maintenance of citizens’ health, as well as increase SPMHCI’s contribution to the improvement of CLS. Firstly, the government could improve its fiscal fund allocation policy and increase investment in primary medical institutions; secondly, based on the actual situation in the region, they could develop reasonable SPMHCI allocation standards and development plans, as well as take steps to meet the needs of SPMHCI training and promotion.

The Chinese government has recently attached great importance to the training and development of SPMHCI. The “China Health and Family Planning Yearbook (2014-2020)” shows that the number of SPMHCI increased from 3.17 million in 2013 to 3.51 million in 2019. This is a very good development. However, there were only 7.27 and 51.89 SPMHCI for every 10,000 urban and rural citizens at the end of 2019. Additionally, about 70.21% of SPMHCI were health technical staff, which is equivalent to 5.10 and 36.43 health technical staff in primary medical and health care institutions for every 10,000 urban and rural citizens, respectively [24]. At the same time, only 37 and 15% of health technicians had a bachelor’s degree or above in urban and rural primary medical and health care institutions [12]. The shortage and lower quality of SPMHCI will result in increased work pressure, lower work quality and easy job burnout [43].

Therefore, firstly, we recommend the government to continue to strengthen medical education by establishing a more reasonable and standardized system of medical student enrollment, education, and training in order to increase the overall number and level of academic qualifications of medical personnel. Secondly, it is necessary to establish and improve the work support system for SPMHCI, and guide them into the development of primary medical and health care institutions using funds and policies. In addition, since China is building medical consortia and medical communities. Medical staff of higher-level medical institutions can use “Internet +” to guide SPMHCI and provide online consultation, disease diagnosis and other services. This strategy will be able to assist SPMHCI in reducing work pressure and increasing their service delivery efficiency [1].

## Limitations

Firstly, the study failed to account for the differences in service efficiency and effectiveness among SPMHCI. Secondly, due to the scarcity of data sources, this study excludes individual clinics from its primary medical and health institutions. In China, individual clinics are not one of the most important components of primary medical and health institutions, and they usually only provide services such as diagnosis of simple diseases and selling medicines. The absence of these particular clinics had no effect on the direction of the primary findings of the current study, however the precise magnitude of the impact will require more investigation once conditions permit. Finally, this study assessed the equity and CCD of SPMHCI concerning quantity and living standards of citizens. It did not attempt to assess the equity of geographical regions. When the difference in population density between regions is small, it is also worthwhile to examine the equity of SPMHCI across geographic regions, hence we hope in our future research to make this consideration a priority.

## Conclusions

This study analyzes the current situation of the population-based Gini coefficient and the CCD between CLS for SPMHCI in China, and predicts the future development trend of equity and CCD to explore the best allocation strategy. The results showed that the number of SPMHCI in China has increased significantly, with equitable population-based allocation, and the national level of equity is better than that of urban and rural areas. At the same time, the interaction between SPMHCI and CLS is sufficient, but the degree of mutual promotion is moderate. As a result, policymakers could take targeted and effective measures to reduce health care spending, such as optimizing SPMHCI allocation and launching chronic disease management services, in order to alleviate the tension between citizens’ health maintenance and living standards improvement.

## Supplementary Information

**Additional file 1:.**

## Data Availability

The data used for this manuscript from China Statistical Yearbook and China Health and Family Planning Yearbook.

## Abbreviations

CCD: Coupling coordination degree
SPMHCI: Staff in primary medical and health care institutions
GM: Grey forecasting model GM (1, 1)
CLS: Citizens’ living standards
