# Supplementary material for: The degree of equity and coupling coordination of staff in primary medical and health care institutions in China 2013–2019
Source: Int J Equity Health. 2021 Oct 30;20:236. doi: 10.1186/s12939-021-01572-6 (PMC8557061; doi:10.1186/s12939-021-01572-6)
Supplement: Supplementary file 1 — Additional file 1:. [file 12939_2021_1572_MOESM1_ESM.doc]

Appendix Table The number of SPMHCI^a^ per 10,000 people in China's urban and rural areas from 2013 to 2019 (people)

| Area | 2013 | | 2014 | | 2015 | | 2016 | | 2017 | | 2018 | | 2019 | |
| --- | --- | --- | --- | --- | --- | --- | --- | --- | --- | --- | --- | --- | --- | --- |
|  | Urban | Rural | Urban | Rural | Urban | Rural | Urban | Rural | Urban | Rural | Urban | Rural | Urban | Rural |
| Total | 6.65 | 42.60 | 6.65 | 43.66 | 6.70 | 44.29 | 6.73 | 46.27 | 6.95 | 48.38 | 7.11 | 49.81 | 7.27 | 51.89 |
| Eastern region^b^ | 7.71 | 46.20 | 7.72 | 46.98 | 7.76 | 47.84 | 7.78 | 49.43 | 8.15 | 51.61 | 8.42 | 52.80 | 8.57 | 54.33 |
| Middle region^c^ | 5.46 | 44.47 | 5.46 | 45.67 | 6.56 | 54.72 | 5.42 | 48.86 | 5.49 | 50.66 | 5.53 | 51.27 | 5.65 | 53.51 |
| Western region^d^ | 5.16 | 39.27 | 5.19 | 40.62 | 5.75 | 44.23 | 5.43 | 43.61 | 5.59 | 46.08 | 5.76 | 48.33 | 6.08 | 50.78 |
| Beijing | 16.62 | 17.23 | 16.51 | 15.32 | 16.73 | 16.45 | 17.45 | 16.41 | 18.73 | 16.20 | 19.95 | 14.78 | 21.02 | 15.95 |
| Tianjin | 6.11 | 42.12 | 5.94 | 45.13 | 6.36 | 44.62 | 6.58 | 44.46 | 7.19 | 47.42 | 7.41 | 48.54 | 7.59 | 49.62 |
| Hebei | 4.10 | 44.06 | 4.27 | 45.38 | 4.19 | 47.43 | 4.11 | 49.85 | 4.23 | 53.03 | 4.43 | 52.78 | 4.45 | 54.94 |
| Shanxi | 6.12 | 44.34 | 6.11 | 44.50 | 5.88 | 45.57 | 5.95 | 47.35 | 6.09 | 49.46 | 6.11 | 50.42 | 6.16 | 52.56 |
| Inner Mongolia | 8.12 | 45.50 | 7.70 | 45.96 | 7.87 | 47.81 | 8.11 | 49.54 | 8.57 | 53.18 | 8.43 | 54.20 | 8.81 | 55.68 |
| Liaoning | 5.12 | 40.84 | 5.23 | 39.00 | 5.36 | 40.03 | 5.61 | 40.81 | 6.32 | 41.67 | 6.37 | 42.48 | 6.42 | 41.62 |
| Jilin | 5.29 | 39.50 | 5.57 | 39.94 | 5.82 | 39.08 | 5.67 | 39.51 | 5.93 | 40.55 | 5.98 | 40.70 | 6.34 | 40.75 |
| Heilongjiang | 7.09 | 34.53 | 7.10 | 34.52 | 6.80 | 35.74 | 6.67 | 36.13 | 6.77 | 36.61 | 6.61 | 36.38 | 6.52 | 36.87 |
| Shanghai | 15.19 | 18.84 | 15.58 | 21.27 | 16.33 | 17.16 | 16.47 | 17.99 | 16.77 | 17.00 | 16.89 | 13.63 | 17.16 | 12.29 |
| Jiangsu | 8.10 | 47.81 | 8.38 | 50.25 | 8.35 | 53.10 | 8.20 | 56.88 | 8.92 | 63.85 | 9.60 | 68.88 | 9.81 | 73.51 |
| Zhejiang | 10.89 | 34.93 | 10.78 | 36.71 | 10.68 | 39.62 | 10.70 | 43.09 | 10.77 | 45.59 | 10.67 | 46.51 | 10.57 | 47.96 |
| Anhui | 6.46 | 38.09 | 6.14 | 38.15 | 5.90 | 38.60 | 5.82 | 39.87 | 5.86 | 41.35 | 5.89 | 41.95 | 5.95 | 43.25 |
| Fujian | 4.85 | 46.70 | 4.83 | 47.47 | 4.84 | 48.68 | 5.06 | 50.85 | 5.39 | 53.32 | 5.54 | 54.78 | 5.66 | 55.74 |
| Jiangxi | 3.95 | 45.58 | 3.83 | 47.49 | 3.74 | 48.82 | 3.54 | 50.33 | 3.28 | 52.54 | 3.15 | 52.77 | 3.28 | 54.85 |
| Shandong | 6.58 | 63.86 | 6.11 | 63.18 | 6.02 | 64.33 | 5.80 | 63.83 | 6.17 | 64.14 | 6.55 | 64.36 | 6.96 | 65.83 |
| Henan | 4.74 | 50.49 | 4.88 | 51.92 | 4.94 | 53.49 | 4.69 | 54.91 | 4.98 | 57.13 | 5.10 | 58.17 | 5.16 | 58.96 |
| Hubei | 6.21 | 50.75 | 6.28 | 54.52 | 6.76 | 56.32 | 6.95 | 59.06 | 6.98 | 60.65 | 6.95 | 62.51 | 6.83 | 62.36 |
| Hunan | 4.33 | 42.15 | 4.41 | 44.42 | 4.38 | 47.25 | 4.63 | 49.54 | 4.64 | 51.87 | 4.95 | 51.65 | 5.45 | 60.73 |
| Guangdong | 6.36 | 38.49 | 6.47 | 37.13 | 6.61 | 37.44 | 6.71 | 39.16 | 6.88 | 40.20 | 7.14 | 41.76 | 7.07 | 42.37 |
| Guangxi | 2.74 | 43.03 | 3.02 | 45.32 | 3.12 | 43.35 | 3.17 | 44.84 | 3.35 | 46.01 | 3.64 | 46.64 | 3.75 | 48.94 |
| Hainan | 5.58 | 37.09 | 5.70 | 39.72 | 5.77 | 42.06 | 5.99 | 43.60 | 6.42 | 46.23 | 5.97 | 49.32 | 6.64 | 50.41 |
| Chongqing | 5.27 | 50.78 | 5.60 | 51.87 | 5.75 | 53.74 | 5.89 | 55.32 | 5.65 | 56.65 | 6.10 | 58.22 | 6.74 | 59.86 |
| Sichuan | 5.12 | 42.45 | 5.07 | 43.84 | 5.02 | 44.38 | 5.31 | 44.85 | 5.28 | 48.27 | 5.33 | 51.66 | 5.43 | 54.25 |
| Guizhou | 4.69 | 33.42 | 4.52 | 35.36 | 5.15 | 39.83 | 5.49 | 43.46 | 6.25 | 42.90 | 6.84 | 44.64 | 7.67 | 48.09 |
| Yunnan | 3.02 | 26.07 | 3.13 | 27.37 | 3.22 | 28.93 | 3.51 | 31.03 | 3.87 | 35.75 | 3.98 | 39.50 | 4.27 | 42.88 |
| Tibet | 1.86 | 57.94 | 1.96 | 64.44 | 2.29 | 67.21 | 2.22 | 65.29 | 2.83 | 73.72 | 2.90 | 77.30 | 2.55 | 79.60 |
| Shaanxi | 5.61 | 45.40 | 5.68 | 45.53 | 5.62 | 46.40 | 5.54 | 49.88 | 5.35 | 51.01 | 5.34 | 54.85 | 5.71 | 56.70 |
| Gansu | 7.07 | 37.51 | 6.91 | 39.30 | 6.95 | 40.61 | 6.69 | 41.83 | 6.76 | 44.29 | 7.20 | 45.11 | 7.51 | 48.24 |
| Qinghai | 7.06 | 48.56 | 7.50 | 49.13 | 7.45 | 50.61 | 7.15 | 48.37 | 8.53 | 54.95 | 8.37 | 56.35 | 8.61 | 59.13 |
| Ningxia | 2.97 | 30.17 | 3.30 | 31.87 | 3.62 | 32.44 | 4.02 | 34.24 | 5.12 | 36.54 | 6.29 | 39.51 | 7.10 | 41.70 |
| Xinjiang | 8.24 | 33.01 | 8.19 | 35.84 | 8.03 | 36.72 | 8.40 | 38.86 | 8.26 | 40.74 | 8.03 | 41.24 | 8.12 | 41.87 |

^a^SPMHCI: Staff in primary medical and health care institutions; ^b^The eastern region includes Beijing, Tianjin, Hebei, Liaoning, Shanghai, Jiangsu, Zhejiang, Fujian, Shandong, Guangdong, and Hainan; ^c^The middle region includes Shanxi, Jilin, Heilongjiang, Anhui, Jiangxi, Henan, Hubei, and Hunan; ^d^The western region includes Inner Mongolia, Chongqing, Guangxi, Sichuan, Guizhou, Yunnan, Tibet, Shaanxi, Gansu, Qinghai, Ningxia, and Xinjiang.
